# Modern Concepts in Precision Targeting of Acute Myeloid Leukemia Based on Principles of Molecular Oncology

**DOI:** 10.3390/cancers18142329

**Published:** 2026-07-19

**Authors:** Robert Yuan, Melissa Mariano, David Cachia, Talha Badar, Shyam A. Patel

**Affiliations:** 1 Division of Hematology/Oncology, Department of Medicine, UMass Memorial Medical Center, UMass Chan Medical School, Worcester, MA 01655, USA; robert.yuan@umassmemorial.org (R.Y.);; 2 Center for Clinical and Translational Science, UMass Chan Medical School, Worcester, MA 01655, USA; 3 UMass CHIP Clinic, Worcester, MA 01655, USA; 4 Bone Marrow Transplant Program, Division of Hematology/Oncology, Mayo Clinic, Jacksonville, FL 32224, USA

**Keywords:** acute myeloid leukemia, precision therapy, synthetic lethality, targeted therapeutics

## Abstract

Acute myeloid leukemia (AML) pathogenesis is deeply rooted in the concept of clonal evolution, in which hematopoietic stem cells experience an initial genetic insult, followed by the sequential acquisition of mutations leading to various disease phenotypes. Through natural selection, a malignant clone can diversify and propagate with time. Aside from clonal evolution, a number of time-honored principles of molecular oncology account for the initiation and maintenance of AML. These principles include loss of heterozygosity, genomic instability, epigenomic disruption, deregulation of signaling pathways, anti-apoptotic mechanisms, and oncogene addiction. Such tenets of cancer biology not only explain AML cellular behavior but also may inform target validation in AML. Herein, we highlight the current state of targeted therapeutics, including the 14 novel agents (beyond 7+3 chemotherapy) approved by the Food and Drug Administration, in the context of these tenets and discuss future prospects by leveraging current knowledge of AML genomics. Such therapeutic concepts, including synthetic lethality, are especially relevant to *TP53*-mutant AML, as there are no unique therapies that have received regulatory approval for this subset to date. We emphasize the emerging role of single-cell genomics and multi-omics toward better understanding of AML biology and development of future successful AML therapies.

## Simple Summary

Acute myeloid leukemia (AML) is a cancer of white blood cells that typically affects older adults. This cancer develops because of genetic mutations in immature stem cells. Over time, as more mutations accumulate in a particular cell, the cell gains the ability to replicate rapidly. There are many mechanisms through which genetic mutations occur. The treatment for AML has changed significantly over the past 50 years, from a one-size-fits-all approach with chemotherapy to a more targeted and individualized plans. Targeted therapies aim to exploit genetic vulnerabilities that make cancer cells more sensitive to these therapies than normal cells. We aim to review the current landscape of precision-targeted therapies in AML through the lens of molecular oncology.

## 1. Introduction

Acute myeloid leukemia (AML) is a biologically heterogeneous hematologic malignancy characterized by the clonal expansion of myeloid progenitors. AML is most common in older adults, with a median age at diagnosis of 68 years. It is a relatively rare cancer, with 22,000 new cases in the United States in 2026, comprising 1.1% of all new cancer diagnoses [1]. With improvements in treatment and supportive care, the 5-year relative survival has improved from 18.5% in 2000 to 33.4% for 2016–2022 SEER data. Historically, AML was treated with intensive chemotherapy comprising cytarabine and an anthracycline, which, while effective, causes significant myelosuppression and treatment-related toxicities in older patients. With significant advances in the understanding of the molecular pathogenesis of AML, there are now multiple targeted therapeutics that offer meaningful benefits in this population.

Our understanding of the genetic basis of AML has grown rapidly in the past 20 years. Once considered a single clinicopathologic diagnosis, AML is now characterized as a group of biologically distinct sub-entities with recurrent genomic aberrations. Mutations affecting epigenetic regulation, signaling pathways, differentiation, apoptosis, and genomic instability all contribute to selecting for and maintaining aggressive clonal leukemic populations and also provide potentially actionable therapeutic targets. In this review, we discuss modern concepts on therapeutic targeting as well as the principles of molecular oncology that form the basis of AML pathophysiology, and we highlight the current state of targeted therapeutics and future prospects on drug development.

## 2. Tenets of Molecular Oncology Governing Myeloid Pathogenesis

### 2.1. Loss of Heterozygosity

**Figure 1:**
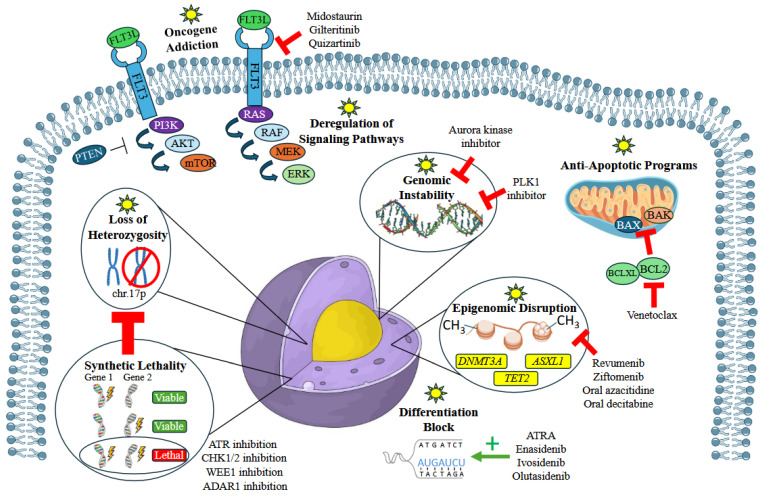
Key principles of molecular oncology involved in AML initiation and maintenance. The major principles governing myeloid pathogenesis are illustrated (yellow stars) in relation to subcellular localization. Synthetic lethality is a therapeutic strategy that may counteract loss of heterozygosity.

One of the major principles of molecular oncology involved in malignant transformation is loss of heterozygosity (LOH) (Figure 1). LOH is a category of genomic aberration in which a cell possessing two different alleles at a genomic locus loses a single allele (copy-loss LOH) and may involve the duplication of the remaining allele (copy-neutral LOH) [1]. The remaining allele is then solely responsible for encoding the resulting protein. In AML, an illustrative example of LOH involves *TP53*, which is the most common gene mutation in human cancer and found in 10–15% of patients with AML [2,3]. *TP53* mutations are frequently seen in cases of AML with complex karyotype, advanced age, and prior exposure to chemotherapeutics [4]. *TP53* is a gene located on chromosome 17p13.1 that encodes for the protein, p53, which is a nuclear transcription factor comprising DNA-binding and transcriptional regulatory domains [5]. p53 is a crucial tumor suppressor that affects hundreds of genes involved in cell cycle arrest or apoptosis [6]. In normal cells, p53 is kept at low levels by murine double minute 2 (MDM2). p53 induces the expression of *MDM2* which in turn promotes the degradation of p53 through ubiquitination [7]. However, when faced with cellular stress, kinases such as Ataxia Telangiectasia Mutated (ATM) and Ataxia Telangiectasia and Rad3-related (ATR) act to phosphorylate p53, stabilizing p53 and inhibiting its interaction with MDM2 [8]. As levels of nuclear p53 increase, p53 can act on downstream genes to facilitate cell cycle arrest and allow for DNA repair or apoptosis.

Mutations in *TP53* that result in abnormal and nonfunctional p53 can occur through multiple mechanisms. Single-allele mutations cause monoallelic *TP53*-mutant AML, whereas copy-loss or copy-neutral LOH, a second *TP53* mutation, or deletion of chromosome 17p can cause biallelic *TP53*-mutant AML. From a clinical standpoint, this has high relevance because “multi-hit” *TP53* is a surrogate for biallelic *TP53* disruption, and this information is used to make informed decisions about patient care. Following acquisition of a single *TP53* mutation, loss of the remaining wild-type *TP53* allele through LOH results in biallelic *TP53* inactivation and complete loss of function of p53, thereby leading to genomic instability. The 2022 European LeukemiaNet (ELN) assigns an adverse risk category to those harboring *TP53* mutations, as it carries a poor prognosis of ~6 months survival due to high rates of treatment failure [9,10]. Recent studies have also shown that while there are distinct co-mutational patterns, some studies have shown no difference in event-free survival or overall survival between single-hit and multi-hit *TP53*-mutated AML [11]. In summary, the oncologic principle of LOH (especially in the case of *TP53*-mutant AML) governs disease biology and has important implications in risk stratification and prognosis, within assay and copy-neutral LOH assessment limitations.

### 2.2. Genomic Instability

Genomic instability is core to the pathogenesis of cancer cell development. Genomic instability in myeloid genomes can arise when a cell acquires an oncogenic mutation, such as *FLT3-ITD*, *RAS*, or *MYC*. The resulting increase in proliferation causes replication stress and further DNA damage [12,13]. These cells then rely on tumor suppressor genes such as *TP53* to cease the cell cycle and induce cell death. If *TP53* function is lost, cells harboring DNA damage can evade cell cycle arrest and apoptosis, allowing propagation of unrepaired DNA lesions and chromosomal defects. The ongoing tendency to rapidly accumulate mutations both at the DNA and chromosomal levels describes the process of genomic instability [14]. In AML, the major form of genomic instability is chromosomal instability (CIN), while other forms like microsatellite instability (MSI) are less common. CIN refers to a high rate at which chromosomal structure and number change resulting from increased chromosome mis-segregation in mitosis [15].

Complex karyotype AML (CK-AML) is defined as having three or more unrelated chromosomal abnormalities in the absence of other class-defining genetic abnormalities and comprises ~10% of all patients with AML [9,16]. The most common abnormalities in CK-AML include loss of 5q, 7q, and 17p [17]. CK-AML is strongly associated with *TP53* mutation, with one study finding that 70% of cases had *TP53* alterations [18]. Another study found that 16 of 17 patients with CK-AML had biallelic *TP53* inactivation [19]. CK-AML is frequently seen in therapy-related AML (t-AML), suggesting that prior stress selects for cells with *TP53* mutations capable of tolerating high rates of genomic instability. CK-AML confers a very poor prognosis, with one study finding median overall survival of 143 days in typical CK-AML and 369 days in atypical CK-AML [20].

### 2.3. Epigenomic Disruption

Epigenetic regulation plays a key role in modifying cellular processes by altering gene expression. The two major mechanisms of epigenetic regulation are DNA methylation and histone modification. DNA methylation occurs when a methyl group is added to a cytosine and in turn recruits proteins involved in gene repression or transcription factor inhibition. DNA methylation mutations can result broadly in hypomethylation or hypermethylation. These most commonly involve *DNMT3A*, *TET2*, and *ASXL1*, which are frequently implicated in clonal hematopoiesis and progression to myelodysplastic neoplasms (MDS) or AML [21].

*DNMT3A* is a DNA methyltransferase gene located on chromosome 2p23, and mutations in this gene often occur in the methyltransferase domain, ultimately leading to aberrant DNA methylation patterns at regions responsible for self-renewal, differentiation block, and expansion of a pre-leukemic clonal population [22]. Missense mutations involving the amino acid R882 are the most common culprits, although frameshift and nonsense mutations also occur. Patients with *DNMT3A* mutations have been shown to experience shorter overall survival compared to those without *DNMT3A* mutations [23].

*TET2* is a ten-eleven translocation gene located on chromosome 4q24 that encodes for a DNA demethylation enzyme. TET proteins oxidize 5-methylcytosine to generate 5-hydroxymethylcytosine, ultimately leading to demethylation [24]. There are multiple *TET* genes, but *TET2* is the most commonly mutated in myeloid malignancies and is present in 10–20% of patients with AML. *TET2* mutations are not independently associated with adverse risk and have not been shown to impact response to therapy or survival [25]. Importantly, mutations in isocitrate dehydrogenase 1/2 (*IDH1/2*) inhibit the TET protein family and affect epigenetic regulation through similar mechanisms [26]. *IDH1/2* and *TET2* mutations are known to be mutually exclusive in myeloid pathogenesis [27].

*ASXL1* is the additional sex combs-like 1 gene and controls epigenetic regulation through histone modification rather than DNA methylation. ASXL1 complexes with BAP1 to facilitate the conversion of chromatin to heterochromatin, regulating transcriptional activity. *ASXL1* mutations occur predominantly through frameshift mutations, and downstream effects include dysregulated chromatin accessibility and increased expression of *HOX* genes, hindering hematopoietic stem cell (HSC) differentiation [28,29]. *ASXL1* mutations are independently associated with poor overall survival and lower response rates to therapy [30]. Together, these mutations illustrate how disruption of the epigenome alters transcriptional programs, thereby contributing to leukemogenesis.

### 2.4. Deregulation of Signaling Pathways

While AML is a genetically heterogeneous disease, individual mutations converge upon a limited number of critical signaling pathways that exhibit significant overlap. These pathways allow for uncontrolled proliferation through complex mechanisms including metabolic reprogramming, aberrant translation, and anti-apoptotic signaling. The key pathways involved include RAF/MEK/ERK, PI3K/AKT/mTOR, and JAK/STAT. In AML, these signaling cascades are more commonly initiated by upstream mutations rather than changes within the signaling mechanisms themselves [31,32]. Mutations in *FLT3*, *KIT*, and *RAS* produce their proliferative effect through activation of several of these pathways at once [33]. The RAF/MEK/ERK and PI3K/AKT signaling network are involved in a variety of cellular processes including cell cycle progression, differentiation, and apoptosis [34]. The PI3K/AKT pathway specifically has been identified as a key mechanism of clonal proliferation in AML, with one study showing 72.1% of adults with untreated AML had constitutive activity of the activating residues [35]. The final major pathway identified is the JAK/STAT signaling cascade. Unlike in myeloproliferative neoplasms where JAK2V617F is the driving mutation, upstream defects, including FLT3 and KIT, are the activating signal. These upstream mutations can constitutively activate STAT signaling, leading to an uncontrolled proliferative signal [36]. Overall, despite the complexity of individual mutations identified in AML, they are funneled into discrete proliferation signaling pathways that drive clonal expansion. These pathways have been difficult to therapeutically target as they exhibit significant overlap and redundancy. Inhibiting one pathway pushes cells to compensate toward reliance on an alternative pathway to gain a survival advantage. Despite strong biologic rationale, direct downstream pathway inhibition in AML has had limited clinical success as compared with targeting upstream lesions such as *FLT3*, *IDH1/2*, or menin-dependent transcription, likely due to extensive pathway crosstalk and compensatory signaling.

### 2.5. Anti-Apoptotic Mechanisms

Apoptosis is an essential mechanism in all cells and allows for orderly cell death in appropriate settings. It allows cells that have reached the end of their life cycle or cells that have experienced irreparable stress to undergo destruction. The normal process involves intrinsic and extrinsic signaling pathways. The extrinsic pathway is triggered when extracellular ligands bind to cell death receptors on the cell’s outer membrane [37]. The intrinsic pathway is activated in response to cellular stress to trigger mitochondrial outer membrane permeabilization (MOMP) and the release of pro-apoptotic factors which subsequently activate caspases and induce apoptosis [38]. This process is tightly regulated by the ratio of pro-apoptotic and anti-apoptotic proteins, which sit on the mitochondrial membrane and set the threshold for apoptosis. BCL-2 is an anti-apoptotic protein that is often overexpressed in AML and inhibits MOMP [39]. As leukemic cells accumulate mutations and become genomically more unstable, they are pushed toward apoptosis through mitochondrial priming [40]. The cells that are able to upregulate BCL-2 avoid apoptosis by restraining pro-apoptotic effector proteins, such as BAX and BAK [37]. Recognition of this dependency led to the development of BCL-2 inhibitors, such as venetoclax, which was approved in 2020 for treatment of AML. Despite this advancement, the presence of other anti-apoptotic proteins may allow leukemic cells to gain adaptive resistance to BCL-2 inhibitors by selecting for populations that depend on other proteins such as MCL-1 or BCL-XL [41]. Consequences of leukemic evolution are gaining recognition, including acquired resistance to venetoclax through selection for monocytic differentiation, increased dependence on alternative anti-apoptotic proteins such as MCL-1 and BCL-XL, and activation of RAS/MAPK pathways.

### 2.6. Oncogene Addiction

Cancer cells accumulate many different genetic mutations that contribute to their survival, but some exhibit intense dependence on a single mutation. The term “oncogene addiction” describes this phenomenon and was first introduced in the early 2000s [42]. In AML, the prototypic example is an activating FLT3 mutation which occur in approximately 30% of AML cases [43]. FLT3 is a transmembrane receptor tyrosine kinase expressed by HSCs. When a FLT3 ligand binds to the receptor, multiple proliferative signaling pathways are activated [44]. There are two forms of FLT3 mutations: internal tandem duplication (ITD) and tyrosine kinase domain (TKD). FLT3-ITD mutations occur most commonly at the juxtamembrane domain where a segment of DNA is duplicated and inserted in tandem, causing constitutive activation [45]. FLT3-TKD is less common and typically results from a point mutation in the kinase domain, changing the conformation and keeping the protein active [46]. The clinical activity of FLT3 inhibitors in relapsed or refractory FLT3-mutated AML provides strong evidence that these phenotypes are dependent on constitutive FLT3 signaling for survival [47,48]. The sensitivity of FLT3-mutant AML to FLT3 inhibitors suggests a state of relative oncogenic addiction to this mutation and provides the biologic rationale for the development of FLT3-targeted therapies.

## 3. Development of Targeted Therapies Based on Principles of Molecular Oncology

### 3.1. Targeting Aberrant Signaling

Midostaurin is a first-generation, type I multi-kinase inhibitor that binds FLT3 and prevents receptor autophosphorylation, thereby disrupting downstream signaling and inhibiting cell proliferation. It has been shown to induce apoptosis in leukemic cells expressing both *FLT3*-ITD and FLT3-TKD mutations [31,32,33] (Table 1). In addition to FLT3, midostaurin inhibits KIT, PDGFR-α/β, VEGFR2, and protein kinase C, resulting in broader kinase activity and potential off-target toxicities.

**Table 1:** Summary of the 14 Contemporary FDA-Approved Agents in AML as of July 2026.

	Mechanism	Clinical Trial Endpoints	Regulatory Approval/Indication	Approval Year and Setting	Adverse Effects	Reference
**Midostaurin**	Type 1 FLT3 inhibitor; also inhibits KIT, VEGFR2, and PDGFR	Improved mOS in combination with 7+3 vs. 7+3 alone (74.7 vs. 25.6 months; HR = 0.78)	Newly diagnosed AML with *FLT3* mutation: 50 mg PO twice daily on days 8–21 during induction and consolidation	2017; Frontline fit	Nausea; increased ALT, hypocalcemia, mucositis	[34] [CALGB 10603/RATIFY]
**Gilteritinib**	Type 1 FLT3 inhibitor	Improved mOS compared with chemotherapy (9.3 vs. 5.6 months), CR rate 21.1%, CR/CRh rate 34.0%	Relapsed/refractory AML with *FLT3* mutation: 120 mg PO daily	2018; R/R	Diarrhea, transaminase elevation, PRES, pancreatitis, QT interval prolongation	[35] [ADMIRAL]
**Quizartinib**	Type 2 FLT3 inhibitor	Improved mOS in combination with 7+3 vs. 7+3 with placebo (31.9 vs. 15.1 months)	Newly diagnosed FLT3-ITD-positive AML: 35.4 mg PO daily on days 8–21 during induction or days 6–19 of HiDAC consolidation, and 26.5 mg PO daily on days 1–14 and 53 mg PO daily thereafter	2023; Frontline fit	QT interval prolongation; torsades de pointes	[38] [QuANTUM-First]
**Enasidenib**	Mutant IDH2 inhibitor	ORR 40%, CR rate 19%, mOS 9.3 months, median duration of response 5.8 months	Relapsed/refractory AML with *IDH2* mutation: 100 mg PO daily	2017; R/R	Hyperleukocytosis, differentiation syndrome, indirect hyperbilirubinemia	[49]
**Ivosidenib**	Mutant IDH1 inhibitor	ORR 41%, CR/CRi rate 30%, CR rate 21%	Relapsed/refractory AML with *IDH1* mutation: 500 mg PO daily	2018; Frontline unfit, R/R	Hyperleukocytosis, differentiation syndrome, QT interval prolongation	[50]
**Olutasidenib**	Mutant IDH1 inhibitor	ORR 48%, CR rate 35%, mOS 11.6 months	Relapsed/refractory AML with *IDH1* mutation: 150 mg PO BID	2022; R/R	Differentiation syndrome, transaminase elevations	[51]
**Revumenib**	Menin inhibitor	CR + CRh 22.8%, ORR 63.2%	Relapsed/refractory acute leukemia with *KMT2A* rearrangement or relapsed/refractory AML with *NPM1* mutation: PO twice daily; dosing varies based on body weight and concomitant CYP3A4 inhibitor use	2024; R/R	Differentiation syndrome, QT interval prolongation	[52] [AUGMENT-101]
**Ziftomenib**	Menin inhibitor	CR + CRh 22%, ORR 33%, mOS 6.6 months	Relapsed/refractory AML with *NPM1* mutation: 600 mg PO daily	2025; R/R	Differentiation syndrome	[53] [KOMET-001]
**CPX-351**	Liposomal cytarabine/daunorubicin; DNA damaging agent	Improved mOS compared to 7+3 (9.5 vs. 5.9 months)	Newly diagnosed t-AML and AML-MRC: fixed dose of 100 mg/m^2^ and 44 mg/m^2^ IV on days 1, 3, and 5 during cycle 1 of induction and days 1 and 3 during cycle 2 of induction; fixed dose of 65 mg/m^2^ and 29 mg/m^2^ IV on days 1 and 3 of consolidation	2017; Frontline fit (secondary AML)	Neutropenic fever, hemorrhage, systolic dysfunction	[54]
**Gemtuzumab ozogamicin (GO)**	Anti-CD33 antibody conjugated to calicheamicin; causes DNA strand scission upon internalization	Improved two-year event-free survival (40.8 vs. 17.1%) and OS (53.2% vs. 41.9%) with addition of GO	Newly diagnosed CD33+ AML: 3 mg/m^2^ IV on days 1, 4, and 7 in combination with 7+3 chemotherapy; monotherapy: 6 mg/m^2^ IV on day 1 and 3 mg/m^2^ on day 8; relapsed/refractory AML: 3 mg/m^2^ IV on days 1, 4, and 7	2017; Frontline fit, R/R, maintenance	Sinusoidal obstruction syndrome, prolonged thrombocytopenia	[55] [ALFA-0701]
**Venetoclax**	BH3 mimetic; BCL2 inhibitor	CR rate 36.7% for 400 mg dose, CR/CRi rate 66.4%, Improved mOS with venetoclax-azacitidine vs. azacitidine (14.7 vs. 9.6 months)	Newly diagnosed AML in combination with azacitidine or decitabine or LDAC for adults ≥ 75 years or who have comorbidities that preclude the use of intensive induction chemotherapy: 400 mg PO daily after ramp-up schedule	2018; Frontline unfit	Diarrhea, hypokalemia, tumor lysis syndrome	[56] [VIALE-A]
**Glasdegib**	SMO/Hedgehog inhibitor	Improved mOS (8.3 vs. 4.3 months) and CR rate (19.2% vs. 2.6%) with glasdegib and LDAC vs. LDAC alone	Newly diagnosed AML in combination with LDAC for adults ≥ 75 years or who have comorbidities that preclude use of intensive induction therapy: 100 mg PO daily on days 1–28 of each 28-day cycle	2018; Unfit, R/R	Alopecia, dysgeusia	[42] [BRIGHT AML 1003]
**Oral azacitidine**	HMA	mOS 24.7 months, median relapse-free survival 10.2 months	Maintenance therapy for adults with AML in first CR/CRi following intensive induction chemotherapy who are not candidates for intensive curative therapy	2020; Maintenance	Vomiting, diarrhea	[57] [QUAZAR AML-001]
**Oral decitabine/** **cedazuridine**	HMA	CR 47%, CR + CRh 63%, mOS 15.5 months	Newly diagnosed AML in adults ≥ 75 years or who have comorbidities that preclude the use of intensive induction chemotherapy: 35 mg decitabine/100 mg cedazuridine PO daily on days 1–5 with venetoclax	2026: Frontline unfit	Vomiting, diarrhea	[58] [ASCERTAIN-V]

Abbreviations: **ALT,** alanine transaminase; **BCL2,** B cell lymphoma 2; **CALGB,** Cancer and Leukemia Group B; **CR,** complete remission; **CRh,** complete remission with partial hematologic recovery; **CRi,** complete remission with incomplete count recovery; **FLT3,** FMS-like tyrosine kinase 3; **IDH,** isocitrate dehydrogenase; **LDAC,** low-dose araC; **ORR,** overall response rate; **mOS**, median overall survival; **PRES**, posterior reversible encephalopathy syndrome; **R/R,** relapsed, refractory; **t-AML,** therapy-related AML.

The pivotal phase III RATIFY trial demonstrated that the addition of midostaurin to standard intensive chemotherapy in newly diagnosed *FLT3*-mutated AML (including both ITD and TKD mutations) significantly improved overall survival and event-free survival. Among patients aged 18 to 60 years, the addition of midostaurin reduced the risk of death by 22% (hazard ratio for death 0.78; one-sided *p* = 0.009) and increased 5-year overall survival, although transplant and censoring likely influenced interpretation of median overall survival difference [34]. Ultimately, this trial led to FDA approval of midostaurin for patients with newly diagnosed *FLT3*-mutated AML.

Gilteritinib is a second-generation, type I FLT3 inhibitor with greater selectivity and potency against the active conformation of FLT3. It induces apoptosis in leukemic cells harboring both FLT3-ITD and FLT3-TKD mutations [33]. Gilteritinib received FDA approval for relapsed or refractory *FLT3*-mutated AML based on the results of the phase III ADMIRAL trial. In this study, gilteritinib significantly improved overall survival compared with salvage chemotherapy (median overall survival, 9.3 months vs. 5.6 months). Gilteritinib also produced higher rates of complete remission and complete remission with partial hematologic recovery compared with salvage chemotherapy (34.0% vs. 15.3%) [35]. In 2024, a phase I/II study evaluating gilteritinib in newly diagnosed *FLT3*-mutated AML patients who were unfit for intensive chemotherapy reported a complete remission rate of 96%, suggesting the potential for expansion of its therapeutic indications [36]. However, although early-phase studies showed promising activity for newly diagnosed *FLT3*-mutant AML, preliminary data from the phase III HOVON trial reported that gilteritinib did not improve overall survival compared with midostaurin-based therapy. Therefore, midostaurin remains the standard FLT3 inhibitor in combination with chemotherapy in the frontline setting, while gilteritinib continues to be standard of care for relapsed or refractory *FLT3*-mutant AML.

Quizartinib is a second-generation, highly selective type II FLT3 inhibitor that binds the inactive conformation of the FLT3 kinase domain, suppressing downstream signaling pathways involved in cell proliferation and survival [37]. Although highly potent against FLT3-ITD mutations, quizartinib has limited activity against FLT3-TKD mutations because these alterations favor the active kinase conformation [33]. The phase III QuANTUM-First trial demonstrated improved overall survival in patients with newly diagnosed FLT3-ITD-positive AML when quizartinib was added to standard induction chemotherapy. Median overall survival was 31.9 months in the quizartinib group compared with 15.1 months in the placebo group, leading to FDA approval of quizartinib in combination with induction and consolidation chemotherapy for newly diagnosed *FLT3*-ITD-positive AML [38].

Several additional studies have investigated the role of quizartinib in AML. The phase III QuANTUM-R trial compared single-agent quizartinib with salvage chemotherapy in relapsed or refractory *FLT3*-ITD-positive AML. Quizartinib improved overall survival compared with chemotherapy, with a median overall survival of 6.2 months versus 4.7 months, respectively [39]. More recently, the phase II QUIWI study evaluated quizartinib in patients with newly diagnosed FLT3-ITD-negative AML with a very low allelic ratio (<0.03). Quizartinib treatment was associated with improved overall survival (60.8% vs. 45.7% at 3 years) and prolonged event-free survival (20.4 vs. 9.9 months; *p* = 0.046) [40]. These findings have led to the ongoing phase III QuANTUM-Wild trial, which aims to confirm the benefits of quizartinib in this patient population.

While FLT3 inhibitors target an oncogenic driver mutation, other therapeutic strategies have focused on signaling pathways that support leukemic stem cell survival and proliferation. One such pathway is the Hedgehog signaling pathway, which works through the transmembrane protein Smoothened (SMO) to ultimately induce expression of genes involved in self-renewal and stem cell maintenance [41]. Leukemic stem cells are often more resistant to chemotherapy than blast cells and rely on the Hedgehog pathway to persist, later contributing to disease relapse. Glasdegib is an oral selective SMO inhibitor that was developed to target these quiescent cells. When combined with low-dose cytarabine, glasdegib was shown in a phase II trial to improve overall survival as well as rate of complete remission in patients with AML or high-risk MDS who were unfit for intensive chemotherapy [42]. However, the follow-up phase III BRIGHT AML 1019 trial did not find a survival benefit with the addition of glasdegib to intensive or non-intensive chemotherapy [43]. While glasdegib is approved in combination with low-dose cytarabine in those unfit for intensive chemotherapy, its use is limited given the negative findings in BRIGHT AML 1019 and the widespread adoption of azacitidine and venetoclax.

### 3.2. Overcoming Differentiation Blockade

Normal HSC differentiation is a highly organized and sequential process. HSCs must balance self-renewal with differentiation into myeloid and lymphoid progenitors. In AML, many mutations can alter this process and create immature clonal populations. Several therapies are available that release leukemic cells to follow their normal differentiation rather than killing the immature cells.

Acute promyelocytic leukemia (APL) is a subtype of AML which is characterized by the PML-RARA fusion protein [44]. This protein, created by the reciprocal t(15;17) translocation, leads to differentiation blockade at the promyelocytic stage. In normal cells, the RARA protein acts as a nuclear transcription factor that controls myeloid differentiation. The PML protein acts as a tumor suppressor by maintaining PML nuclear bodies, which mediate interactions between products of cellular stress, including p53. When these proteins are fused together, they create a gain-of-function oncogene that actively represses differentiation genes. The RARA protein is no longer able to facilitate differentiation and the PML protein cannot mediate apoptosis.

Since its introduction as differentiation therapy for APL in 1988, all-trans retinoic acid (ATRA) has remained the cornerstone of treatment [45]. ATRA binds to the PML-RARA fusion protein and causes it to dissociate from corepressor complexes to reactivate differentiation genes, thereby restoring genes required for granulocyte differentiation [46]. Tallman et al. showed superior rates of disease-free and overall survival with the incorporation of ATRA into induction and maintenance therapy compared with a chemotherapy-based approach [47]. Modern APL treatment was established by the landmark study by Lo-Coco et al., who showed significantly improved two-year event-free survival rates of 97% with the addition of arsenic trioxide to ATRA compared to 86% for those who received ATRA and chemotherapy [48]. The addition of arsenic also improved the probability of 2-year overall survival from 91% to 99%, while reducing hematologic and infectious complications. More recently, the phase III APOLLO trial showed improved rates of event-free survival, molecular relapse after complete remission, and fewer adverse events with ATRA-ATO when compared with ATRA with anthracycline-based chemotherapy in high-risk APL [59].

The most important complication of ATRA and arsenic is differentiation syndrome (DS), which is characterized by fever, respiratory distress, edema, hypotension, among other life-threatening conditions [60]. It originates from differentiation-driven pulmonary cytokine production and recruitment and adhesion of inflammatory cells causing endothelial activation and capillary leak [61,62]. Many centers incorporate preventative approaches with corticosteroids, especially in patients with high leukocyte levels (>5–10 × 10^9^/L) [63]. Treatment of overt DS is with corticosteroids and supportive care.

Although APL remains the prototypic example of differentiation targeted therapies, differentiation blockade is not unique to APL. Similar principles have guided targeted treatments in *IDH1/2*-mutated AML. Mutations in *IDH1* and *IDH2* are common and occur in ~20% of patients with AML [64]. These gain-of-function mutations affect the Krebs cycle and cause the abnormal reduction of alpha-ketoglutarate to 2-hydroxyglutarate (2-HG). The production of 2-HG inhibits de-methylation enzymes such as TET, leading to a hypermethylated state and repression of differentiation programs.

Several *IDH1/2* mutation-directed therapies have been developed to release this differentiation blockade. Enasidenib is a selective inhibitor of mutant IDH2 and was the first IDH2-targeted treatment developed. Stein et al. demonstrated that enasidenib lowered in vivo plasma 2-HG and promoted myeloid differentiation [49]. Treatment with enasidenib also led to DS in approximately 10% of patients. Soon after, DiNardo et al. showed similar findings of durable differentiation-based clinical responses for the mutant IDH1 inhibitor ivosidenib [50]. The phase III AGILE trial evaluated the addition of ivosidenib to azacitidine for patients with *IDH1*-mutated AML who were ineligible for induction chemotherapy. They found that the addition of ivosidenib significantly improved complete response rates, event-free survival, and overall survival. These findings established mutant IDH1 inhibition as a frontline therapeutic strategy and demonstrated that reversal of the differentiation blockade could translate to meaningful survival benefits. Further evidence was provided by olutasidenib, another highly selective IDH1 inhibitor, which demonstrated efficacy in the relapsed/refractory population and among those who had previously received ivosidenib [51].

### 3.3. Targeting Epigenetic Dysregulation

Mutations involving epigenetic dysregulation are some of the earliest mutations in clonal hematopoiesis of indeterminate potential (CHIP) and MDS and are recognized as hallmarks of AML. They create a molecular environment primed for self-renewal and clonal expansion. Hypomethylating agents (HMA) were developed to broadly target the epigenetic programming to return to a normal state of differentiation. The first HMA was azacitidine, which is a cytidine nucleoside analog that incorporates into RNA and DNA to form irreversible bonds with DNA methyltransferases. This results in degradation of DNMT and remodeling of abnormal transcriptional programs to promote differentiation and, importantly, to sensitize cells to other therapies [65].

Azacitidine was initially studied in MDS, where it showed great promise in increasing overall survival. In the phase III trial by Fenaux et al., one-third of their study population was later considered to have had AML, which led the authors to investigate azacitidine in elderly patients with AML. Up to that point, treatment options for older patients or those unfit for intensive chemotherapy were limited, which posed a difficult problem as the average age at diagnosis is 69 years old. The authors found that azacitidine significantly increased median overall survival as well as 2-year overall survival rates. This represented a treatment paradigm shift, as older and unfit patients were now eligible for relatively effective cancer-directed therapy. Decitabine is another HMA that showed similarly meaningful response rates and acceptable tolerability in elderly AML populations. Despite these advancements, outcomes with HMA monotherapy remained modest. In 2020, the AML treatment landscape changed when VIALE-A demonstrated high remission rates and meaningful survival improvements with the combination of azacitidine and venetoclax compared with azacitidine and placebo in newly diagnosed patients with AML ineligible for intensive chemotherapy. This trial changed the treatment approach by introducing azacitidine as the backbone of therapy, paving the way for its combination with many other targeted therapies. A recent phase 2b trial evaluated the fixed-dose all-oral combination of decitabine-cedazuridine plus venetoclax for patients with newly diagnosed AML who were unfit for intensive chemotherapy. The decitabine-cedazuridine combination achieves systemic decitabine exposure comparable to intravenous administration, and the regimen produced CR rates of 47%, CR/CRi rates of 63%, and a mOS of 15.5 months [58]. An all-oral regimen has the potential to significantly improve patient quality of life and caregiver burden.

One such combination includes menin inhibitors, which constitute the most modern targeted therapies. Menin is encoded by the gene *MEN1* and functions as a chromatin-associated scaffold protein involved in transcriptional regulation. In AML pathogenesis, menin facilitates the interaction between mutated KMT2A fusion proteins and chromatin. The result is constitutive activation of the oncogenes, *HOXA9* and *MEIS1* [66]. Menin also plays a crucial role in *NPM1*-mutated AML, where the abnormal NPM1 protein utilizes the normal KMT2A-menin complex to sustain *HOXA9/MEIS1* expression [67]. NPM1 is the most common mutation in AML, present in up to 30% of cases [68]. The KMT2A-menin interaction is essential for maintaining the leukemogenic transcriptional program in both *KMT2A*-rearranged and *NPM1*-mutated AML, facilitating the self-renewal of undifferentiated leukemic stem cells. Pharmacologic disruption of this interaction suppresses this transcriptional program and promotes myeloid differentiation and leukemic cell death. Revumenib was the first-in-class oral inhibitor of the menin-KMT2A interaction and was shown to have high remission rates in relapsed and refractory *KMT2A*-rearranged and *NPM1*-mutated AML [52]. Ziftomenib also showed similar clinical benefits in heavily pretreated relapsed and refractory *NPM1*-mutated AML [53]. Collectively, these studies established menin inhibitors as a novel therapeutic strategy that exploits shared epigenetic and transcriptional dependencies in *KMT2A*-rearranged and *NPM1*-mutated AML. Revumenib has FDA approvals in relapsed/refractory AML with susceptible *NPM1* or *KMT2A* aberrations. Ziftomenib is approved for adults with relapsed/refractory AML with susceptible *NPM1* mutations.

### 3.4. Counteracting Anti-Apoptotic Mechanisms

AML cells are highly dependent on anti-apoptotic mechanisms. Measuring mitochondrial apoptotic sensitivity using BH3 profiling, Vo et al. showed that degree of mitochondrial priming correlated with treatment response, introducing BCL-2 inhibition as a potential novel therapeutic class [69]. Two years later, the BCL-2 inhibitor ABT-199 (now venetoclax), showed efficacy in mitochondrial targeting and cell death, prompting further clinical testing [70]. When utilized for unfit patients or those with relapsed/refractory disease, venetoclax monotherapy demonstrated a modest response rate and acceptable toxicity [71]. Given its limited efficacy alone, venetoclax was combined with other therapies, in hopes of producing a synergistic effect. Azacitidine had become a commonly used backbone for combinations given its efficacy and tolerability. The biologic rationale was that altering the epigenetic environment increased mitochondrial priming and dependence on BCL-2-mediated survival, thereby sensitizing cells to venetoclax-induced apoptosis. Then, when venetoclax inhibits the anti-apoptotic function of BCL2, these mitochondrially primed cells are able to undergo apoptosis. The VIALE-A trial established the combination of azacitidine and venetoclax as the preferred standard-of-care for newly diagnosed AML patients who were elderly or otherwise unfit for intensive chemotherapy [56]. Despite its transformative impact on AML treatment, primary and acquired resistance to venetoclax remains a significant challenge and is frequently driven by cellular adaptation and reliance on alternative anti-apoptotic pathways. Efforts to overcome these escape mechanisms are ongoing and include novel combination strategies and triplet regimens.

### 3.5. Targeting Cell Surface Antigens

CD33 is a transmembrane glycoprotein expressed on the surface of myeloid progenitors and mature cells of the myeloid lineage. CD33 is downregulated as myeloid cells mature and is largely absent from HSCs and nonhematopoietic cells and thus serves as an attractive therapeutic target in AML. Most (90%) of patients with AML are CD33-positive and efforts to target this receptor resulted in the development of gemtuzumab ozogamicin, an antibody-drug conjugate consisting of an anti-CD33 monoclonal antibody linked to the cytotoxic agent calicheamicin [72]. In early trials, gemtuzumab ozogamicin monotherapy demonstrated a relatively favorable safety profile [73]. Despite initially promising results, interim analysis from the SWOG S0106 study that evaluated the addition of gemtuzumab ozogamicin to induction or maintenance therapy did not show an improvement in rates of remission and showed an increase in serious induction toxicity, such as hepatic sinusoidal obstruction syndrome (SOS), leading to the temporary withdrawal of the agent from the market [74]. The ALFA-0701 study shortly after revealed that using fractionated lower doses of gemtuzumab ozogamicin allowed for safer and higher cumulative dose delivery and significantly improved outcomes when added to standard induction treatment [55]. Further identification of the optimal patient population was established through a meta-analysis which found that survival benefits were most pronounced in the favorable and intermediate risk category [75]. Currently, gemtuzumab ozogamicin is approved for use in newly diagnosed CD33-positive AML in combination with standard induction chemotherapy, as monotherapy in those unfit for intensive chemotherapy, in relapsed or refractory disease, and as continuation therapy.

### 3.6. Exploiting Synthetic Lethality

Synthetic lethality describes the interaction between two genes whereby when one gene is mutated, the cell is able to survive, but when both genes are dysfunctional, the cell dies [76]. The cell depends on the non-mutated gene to accomplish functions essential to cell survival to compensate for the mutated gene. This vulnerability was first targeted in human cancers in 2005 when it was shown that *BRCA1/2*-mutant cells were highly dependent on normal PARP1 activity. BRCA proteins are essential tumor suppressors that repair DNA double-stranded breaks by homologous recombination. PARP1 proteins also work to repair DNA damage, although through repair of single-strand breaks. Two studies in 2005 showed that *BRCA1/2*-deficient cell lines were highly dependent on PARP1 activity and the inhibition of PARP1 led to cell cycle arrest and apoptosis [77,78]. Importantly, synthetic lethality represents a potentially tumor-specific therapy, as normal cells with two functional proteins would be spared from cell death. The introduction of large-scale next-generation sequencing has expedited and simplified the process of identifying genetic interactions. As AML cells accumulate mutations, they become increasingly dependent on alternative pathways, creating new vulnerabilities. The loss of one component of the cellular machinery shifts that role to a backup mechanism. Unlike oncogene addiction, which exploits dependence on a gain-of-function mutation, synthetic lethality arises from dependencies created from a loss-of-function event.

Synthetic lethality has emerged as a promising new therapeutic strategy in AML, although no such treatments have yet received regulatory approval. Mutant *IDH1* and *IDH2* produce 2-HG, which leads to decreased DNA double-stranded break repair through decreased ATM function [79]. As a result, leukemic cells become increasingly reliant on PARP-dependent DNA repair pathways for survival. AML cells with IDH mutations were sensitive to the PARP inhibitors, olaparib and talazoparib, in preclinical trials [79,80]. Ongoing clinical trials, including the PRIME trial, are testing the effectiveness of olaparib in *IDH1/2*-mutant relapsed and refractory AML and MDS [81]. Another approach involved the use of BCL-2 inhibitors in *IDH1/2*-mutant AML [82]. *TP53*-mutant AML is particularly well suited to synthetic lethality therapeutic approaches. In the setting of *TP53* mutation, the leukemic cells increasingly accumulate genomic instability and replication stress. These cells heavily rely on compensatory DNA damage response and cell-cycle checkpoint pathways to maintain viability. Therapeutic inhibition of these backup pathways is an attractive mechanism through which to selectively induce leukemic cell death in this ultra high-risk AML subtype. A very recent study showed the value of decitabine plus ATR inhibition (as a synthetic lethal pair) in *TP53*-mutant AML [83]. Another recently discovered hit is ADAR1 in *TP53*-mutant AML, as ADAR1 loss appears to result in exquisite sensitivity in the setting of dysfunctional *TP53* status [84]. Other synthetic lethality- approaches in AML include the use of WEE1 inhibition with adavosertib in *TP53*-deficient AML [85]. In these cells, *TP53* mutation leads to loss of the G1 checkpoint and reliance on WEE1-mediated G2 arrest. Inhibiting WEE1 leads to mitotic catastrophe and cell death. Collectively, these approaches represent the potential of synthetic lethality to expand the repertoire of targeted therapies in AML.

## 4. Conclusions and Future Outlook

### 4.1. Single-Cell Genomics Toward Resolving Clonal Architecture in AML

The introduction of next-generation sequencing (NGS) testing in AML has led to the ability to more precisely risk-assess patients and has also led to the ability to carefully select targeted therapeutics when appropriate. However, the major downside to conventional NGS technologies for AML appraisal is that the assay is often performed in bulk on bone marrow specimens and does not have the ability to resolve the clonal architecture on a cell-by-cell basis. This limitation of bulk sequencing has important implications in AML prognostication and management, as AML is a highly heterogeneous disease defined by baseline mutations and susceptible to on-treatment clonal evolution within a given patient. The lack of the ability to perform single-cell sequencing prevents a clinician from understanding the plasticity of AML, which may change during each patient’s treatment journey. Single-cell assays may be promising research tools rather than tools in near-term routine clinical practice, given cost, turnaround time, standardization, and reimbursement barriers.

Future research would benefit from exploring these aspects of single-cell genomics, which have significant implications in the future of AML diagnosis, prognostication, and treatment [86]. Characterization of individual cells is especially important for patients with suspected biallelic *TP53* mutation, as even a very small burden of biallelic *TP53*-mutant cells may result in eventual relapse or contribute to refractory disease. Single-cell DNA sequencing would be able to assess for biallelic mutations (and determine the allelic state for particular gene mutations), and single-cell RNA sequencing would help assess the functional genome through appraisal of transcripts. Single-cell genomics may be particularly useful for MRD assessment, as MRD is a major predictor of clinical outcomes and eventual disease relapse [87]. Scalability is currently a barrier, as the cost for performing and standardizing single-cell sequencing can be prohibitive in resource-limited medical centers.

### 4.2. Multi-Omics Toward Strategic Target Validation in AML

Contemporary assessments for AML typically involve cytogenetic analyses (for gross structural abnormalities) and gene mutational analyses. Future prospects in AML disease assessment extend beyond basic genomics; such assessments may include appraisal of the epigenome, transcriptome, proteome, cell surfaceome, and the recombinome in select patients. The integration of these techniques may help with precision prognostication and possibly therapeutics in the future. Multi-omics can shed insight into disease behavior and functional implications of genomic aberrations, as it can provide a 3-dimensional snapshot of the key functionalities of the cell [88]. Multi-omics may assist with discerning between causation and correlation, which is important in the field of AML as many recurrent mutations are often passenger mutations rather than driver mutations. Multi-omics might also assist with discerning between pre-leukemic clonal hematopoiesis, which is often harmless in the correct clinical context, and pathogenic hematopoiesis. In 10–20% of patients with AML, the clinical ontogeny is related to prior therapy exposure (therapy-related), and multi-omics might be able to determine the true ontogeny with regard to genotoxic stress imparted by the prior exposure [89]. It may also assist in clonal dynamic modeling for better ascertainment of the disease [90].

Within the realm of multi-omics for AML lies optical genome mapping, which is an emerging technique that is being studied to bypass the limitations of conventional cytogenetics. Optical genome mapping can provide high-resolution assessment of cryptic structural variants or copy-number variations and might relay information about other cellular processes [91,92,93]. This might have the most benefit in AML subtypes driven by rare genomic abnormalities or in AML subtypes characterized by significant structural changes such as *TP53*-mutant AML. Optical genomic mapping has the ability to detect chromoanagenesis and changes in DNA ploidy, which has clinical implications for responsiveness versus refractoriness to chemotherapy. In a multi-center study of optical genomic mapping for 100 patients with AML, it was found that this technique was able to identify important information in 13% of cases that had not been detected by conventional cytogenetics [91]. In some cases, cryptic translocations can be uncovered with optical genomic mapping in a seemingly normal bone marrow aspirate. The diagnostic utility of optical genomic mapping may improve in the future as additional data accrues. However, the technique still requires validation, viable sample/input DNA quality, workflow integration, and correlation with conventional cytogenetics and NGS.

It is also important to recognize limitations associated with precision targeting through multi-omics in AML. Although there are numerous benefits to multi-omics approaches toward precision targeting in AML, enthusiasm may need to be tempered because multi-omics faces the challenges of unknown data integrity, gaps in integration of data sources, high operational costs, and lack of clinical validation in AML. As for data integrity and integration gaps, the sources of raw data in AML multi-omics might not be uniform across platforms, and heterogeneous data sets can introduce some scientific error. Examples may include diverse detection limits for molecular signals (such as MRD monitoring) and diverse methods for measurement of mutations in AML. Furthermore, the biologically meaningful results might be shadowed by background noise in the setting of highly complex data on AML epigenetics, genomics, transcriptomics, and proteomics [94]. This is especially true for massive datasets in which a number of data points may interfere with relevant conclusions. Operational costs are likely to be high given the sophistication in methodologies required for comprehensive appraisal of leukemia cells, and the high cost would subsequently pose issues with accessibility in community oncology practices or in non-specialized treatment centers. Finally, since multi-omics is still within the investigational phase, the field lacks clinical validation in patient datasets, so additional work remains to be done in pilot cohorts, followed by larger registries [95].

Perhaps the most important role for AML multi-omics lies within biomarker discovery toward development of novel therapies, or at least selection of existing therapies in the ideal clinical context. Multi-omics may provide information about molecular target validation, typically including targets on the cell surface as well as intracellular targets, on a patient-by-patient basis. For patients with medically complex subtypes of AML, multi-omics might also pave the way for precision selection of therapeutics against the most dominant clone at a given timepoint during the patient’s journey. The application of multi-omics across human disease can pay major dividends in the coming years, especially for diseases characterized by high biological diversity such as AML.

## Data Availability

No new data were created or analyzed in this study. Data sharing is not applicable to this article.
